# Supplementary figures and images for: OsBTBZ1 Confers Salt Stress Tolerance in Arabidopsis thaliana
Source: Int J Mol Sci. 2023 Sep 23;24(19):14483. doi: 10.3390/ijms241914483 (PMC10572369; doi:10.3390/ijms241914483)

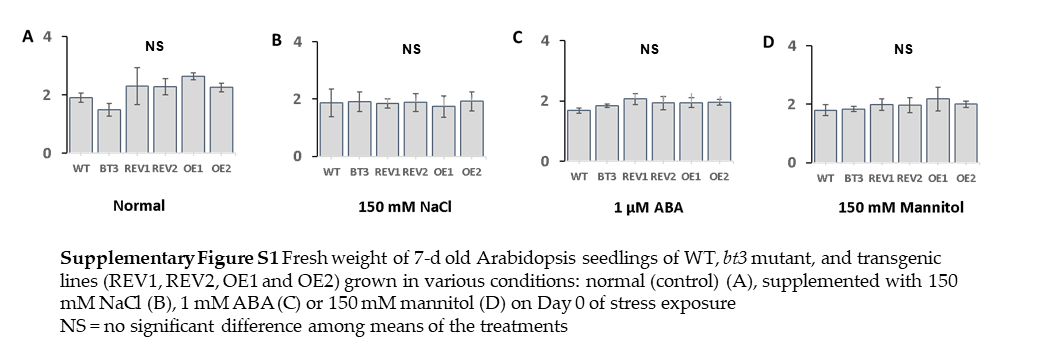

Supplement: Supplementary file 1 [file ijms-24-14483-s001.zip › Supplementary Figure S1.TIF]

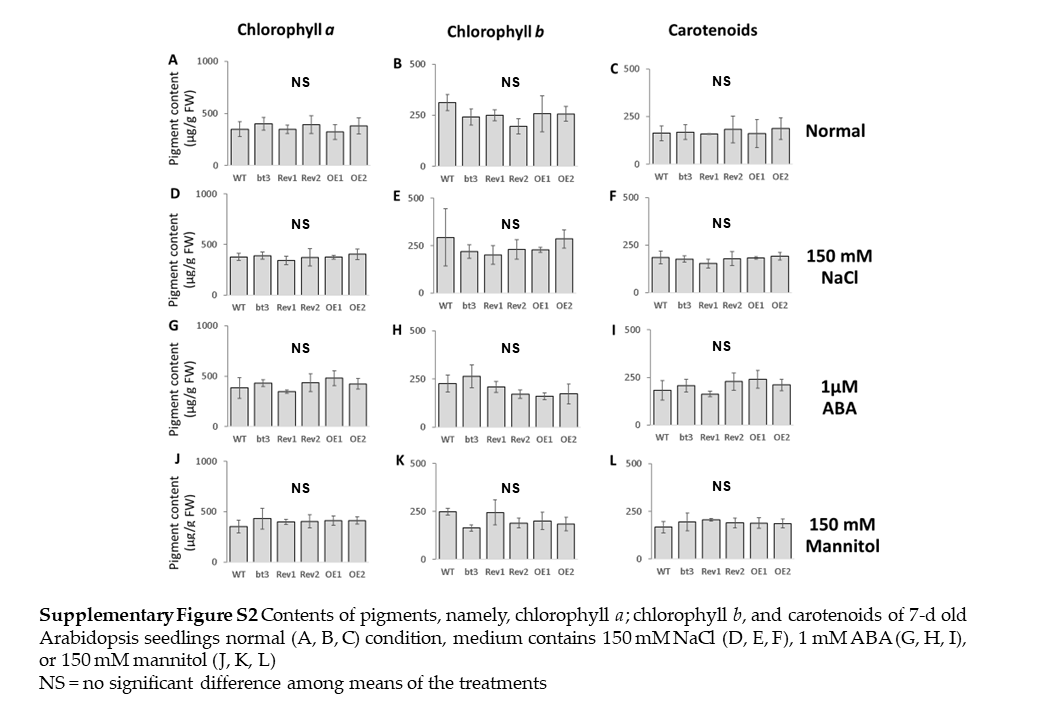

Supplement: Supplementary file 1 [file ijms-24-14483-s001.zip › Supplementary Figure S2.TIF]

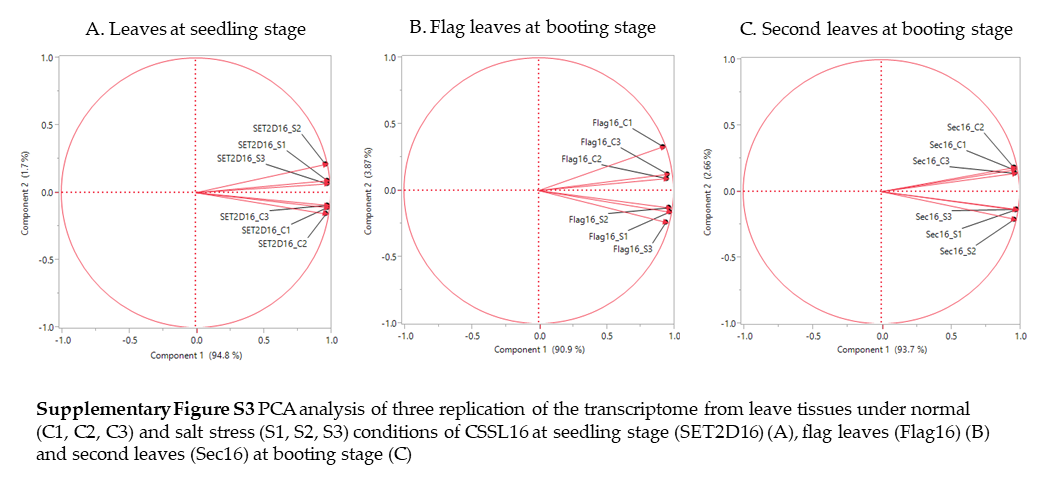

Supplement: Supplementary file 1 [file ijms-24-14483-s001.zip › Supplementary FigureS3.TIF]

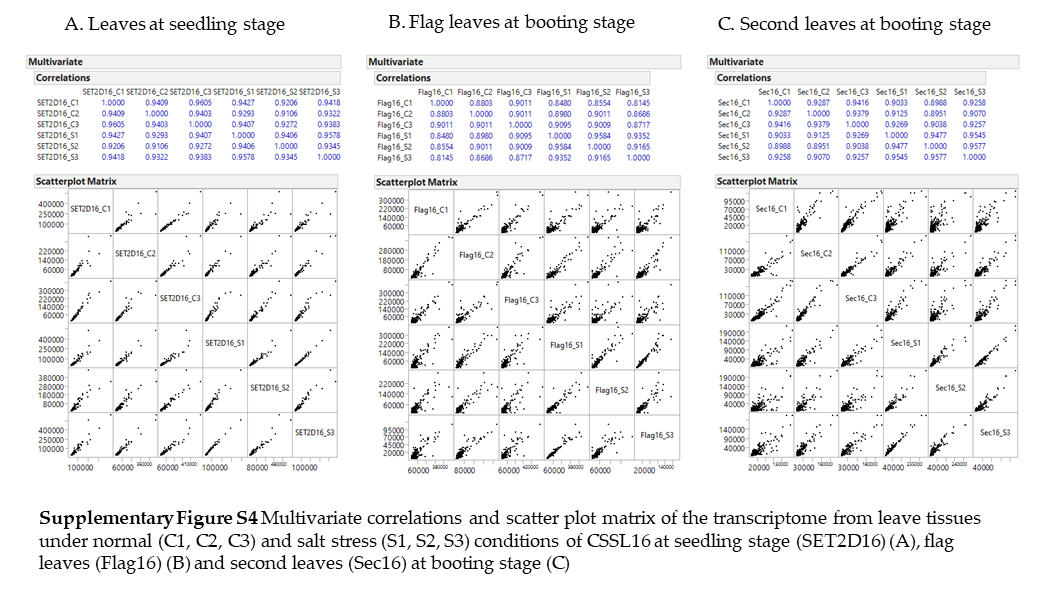

Supplement: Supplementary file 1 [file ijms-24-14483-s001.zip › Supplementary FigureS4.TIF]

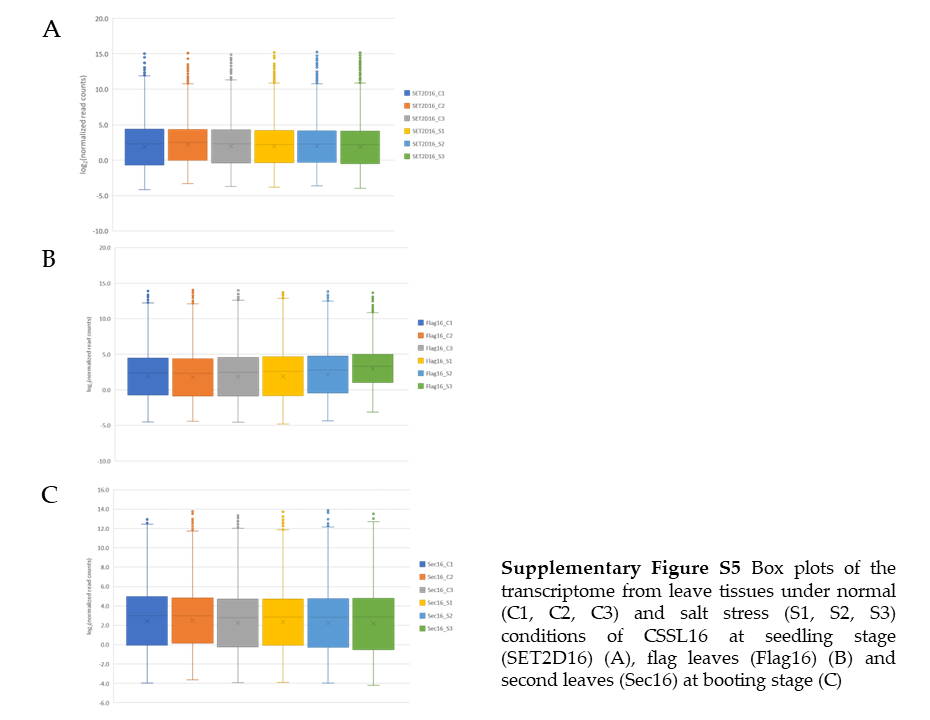

Supplement: Supplementary file 1 [file ijms-24-14483-s001.zip › Supplementary FigureS5.TIF]
